# Signaling Complexity Measured by Shannon Entropy and Its Application in Personalized Medicine

**DOI:** 10.3389/fgene.2019.00930

**Published:** 2019-10-21

**Authors:** Alessandra J. Conforte, Jack Adam Tuszynski, Fabricio Alves Barbosa da Silva, Nicolas Carels

**Affiliations:** ^1^Laboratory of Biological Systems Modeling, Center of Technological Development in Health, Oswaldo Cruz Foundation, Rio de Janeiro, Brazil; ^2^Laboratory of Computational Modeling of Biological Systems, Scientific Computing Program, Oswaldo Cruz Foundation, Rio de Janeiro, Brazil; ^3^Department of Oncology, University of Alberta, Edmonton, AB, Canada; ^4^Department of Physics, University of Alberta, Edmonton, AB, Canada; ^5^DIMEAS, Politecnico di Torino, Turin, Italy

**Keywords:** molecular target, precision medicine, chemotherapy, RNA-seq, interactome

## Abstract

Traditional approaches to cancer therapy seek common molecular targets in tumors from different patients. However, molecular profiles differ between patients, and most tumors exhibit inherent heterogeneity. Hence, imprecise targeting commonly results in side effects, reduced efficacy, and drug resistance. By contrast, personalized medicine aims to establish a molecular diagnosis specific to each patient, which is currently feasible due to the progress achieved with high-throughput technologies. In this report, we explored data from human RNA-seq and protein–protein interaction (PPI) networks using bioinformatics to investigate the relationship between tumor entropy and aggressiveness. To compare PPI subnetworks of different sizes, we calculated the Shannon entropy associated with vertex connections of differentially expressed genes comparing tumor samples with their paired control tissues. We found that the inhibition of up-regulated connectivity hubs led to a higher reduction of subnetwork entropy compared to that obtained with the inhibition of targets selected at random. Furthermore, these hubs were described to be participating in tumor processes. We also found a significant negative correlation between subnetwork entropies of tumors and the respective 5-year survival rates of the corresponding cancer types. This correlation was also observed considering patients with lung squamous cell carcinoma (LUSC) and lung adenocarcinoma (LUAD) based on the clinical data from The Cancer Genome Atlas database (TCGA). Thus, network entropy increases in parallel with tumor aggressiveness but does not correlate with PPI subnetwork size. This correlation is consistent with previous reports and allowed us to assess the number of hubs to be inhibited for therapy to be effective, in the context of precision medicine, by reference to the 100% patient survival rate 5 years after diagnosis. Large standard deviations of subnetwork entropies and variations in target numbers per patient among tumor types characterize tumor heterogeneity.

## Introduction

Statistical and epidemiological data indicate that cancer is a growing global health problem. The World Health Organization (WHO) predicts an estimated 27 million new cases of cancer worldwide by 2030. Cancer initiation and progression involves genetic and epigenetic changes that reprogram complex regulatory circuits. Within this context, Hanahan and Weinberg (Hanahan and Weinberg, 2011) characterized 10 consensus processes, called cancer hallmarks, which are representative of oncogenesis.

Traditionally, a protocol of chemotherapy is considered beneficial for an entire patient subpopulation with common tumor traits and is, therefore, referred to as one-size-fits-all. However, molecular diversity increasing with tumor development promotes therapy resistance (van Wieringen and van der Vaart, 2011; Banerji et al., 2015). Moreover, chemotherapy drugs may result in harmful side effects for patients due to their low selectivity that adversely affects both tumor and normal cells (Siegel et al., 2012). Thus, the process of therapeutic target identification is complex and implies the recognition of molecular differences between tumor and healthy cells, most of them based on gene regulation. Accordingly, the profile of up-regulated genes in tumor tissues is used in a personalized (individualized) medicine approach. Personalized medicine is expected to bring higher benefits to patients.

The development of personalized medicine is directly related to high-throughput technologies that became available in recent years. High-throughput techniques, such as RNA sequencing, are important tools for the characterization of tumor and control cells. These techniques allow a better understanding of tumor biology and demonstrate that each tumor is unique.

Many efforts are being made to identify new targets that could assist in individual treatment. An approach recently used was the identification of five specific therapeutic targets for each of seven breast cell lines (Carels et al., 2015a). This strategy combines protein–protein interactions (PPI) and RNA-seq data to infer the topology of the regulatory network for each cell line. Three concepts were considered in this approach: i) a vertex with a high expression level is more influential than a vertex with a low expression level; ii) a vertex with a high connectivity level (hub) is more influential than a vertex with a low connectivity level; and iii) a protein target must be expressed at a significantly higher level in tumor cells than in control cells to reduce harmful side effects to the patient after its inhibition. It is worth mentioning that each combination of targets that most closely satisfied these conditions was specific for its respective cell line.

This approach was validated experimentally *in vitro* in MDA-MB-231 (a triple-negative cell line of invasive breast cancer) (Tilli et al., 2016) and showed that the inactivation, by interference RNA, of the five top-ranked targets identified for this cell lineage resulted in a significant reduction of cell proliferation, colony formation, cell growth, cell migration, and cell invasion. Inactivation of these targets in other cell lines, such as MCF-7 (non-invasive breast cancer) and MCF-10A (control), showed little or no effect, respectively (Tilli et al., 2016). In addition, the effect of joint target inactivation was greater than the one expected from the sum of individual target inhibitions, which is in line with the buffer effect of regulatory pathway redundancy in tumor cells.

Inactivating multiple hubs may be necessary to shut down alternative pathways that maintain the tumor malignancy. Other authors have also shown that the use of combined drugs is more efficient than monotherapies (Fumiã and Martins, 2013).

The analysis of signaling pathways as networks has been widely used to explore the synergistic effect of targeting multiple proteins and for identifying new targets for cancer treatment. Topological measures regarding node centrality, degree, and path metrics have been used in the identification of regulation patterns and new potential targets for cancer treatment (Schramm et al., 2010; Peng et al., 2014). For instance, Azevedo and Moreira-Filho (Azevedo and Moreira-Filho, 2015) used node degree and betweenness centrality measures to explore the synergistic effects of potential target combinations to overcome chemotherapy resistance to temozolomide in glioma. The network robustness after node removal was assessed considering the following network parameters: diameter, shortest path length, size, and the clustering coefficient. Winterbach et al. (Winterbach et al., 2013) reviewed network metrics and types to discuss their applications and limitations in descriptive and predictive network analyses.

The signaling network of a biological system is scale-free (Albert et al., 2000), which means that few proteins have high connectivity values and many proteins have low connectivity values. As a consequence, the inhibition of proteins with high connectivity values has a greater potential for network disruption than randomly selected proteins (Albert et al., 2000).

The impact of node removal can also be evaluated by the use of Shannon entropy, which has been proposed as a network complexity measure and applied by many authors to determine a relationship between network entropy and tumor aggressiveness. Breitkreutz et al., for instance, found a negative correlation between the entropy of networks composed by genes documented in the Kyoto Encyclopedia of Genes and Genomes (KEGG) database considering cancer types and their respective 5-year survival (Breitkreutz et al., 2012).

Other studies adapted the Shannon entropy formula to combine a unique signaling network and multiple transcriptome data related to the considered phenotypes. Wieringen and Vaart (van Wieringen and van der Vaart, 2011) found that, when considering transcriptome data, the entropy level of cancer samples is higher than that of normal samples. The same behavior was found considering tumor stages, where more advanced stages were characterized by higher entropy than the earlier ones (Breitkreutz et al., 2012; Winterbach et al., 2013; Banerji et al., 2015).

The Shannon entropy is calculated according to formula 1 below (Shannon, 1948) and allows the quantification of information content associated with the likelihood that a given vertex may have a given connectivity value in the considered network. The Shannon entropy (*H*) is given by the formula

(1) $$H=-\Sigma_{k=1}^{n}p(k)\log_{2}(p(k))$$

where *p*(*k*) is the probability that a vertex with a connectivity value (*k*) occurs in the analyzed network. Since entropy is an extensive thermodynamic function of states, it should not be normalized for network size.

In this report, we considered the concepts of connectivity hubs, up-regulated genes, and Shannon entropy in order to assess tumor complexity and to infer a personalized medicine approach. We used the transcriptome data from tumors and their paired non-tumoral tissue considered as control samples to determine their up-regulated genes, construct their corresponding subnetworks, and calculate their respective entropy. We performed this exercise individually for the data collected from 475 patients distributed among nine cancer types. The results confirmed the existence of a negative correlation between the entropy of a tumor’s PPI subnetwork and the corresponding survival rate using data from bench experiments (The Cancer Genome Atlas, TCGA). We also propose a method to infer the suitable number of targets for inhibition according to the 100% patient survival 5 years after treatment. This method concerns the number of connectivity hubs that should be inactivated in a tumor to lower its subnetwork entropy to a level that maximizes patient survival.

To our knowledge, this is the first report aiming at the application of Shannon entropy, transcriptome data, and individual signaling subnetwork mining in the design of a personalized approach for cancer therapy.

## Materials and Methods

### Gene Expression Data

The gene expression data were obtained as RNA-seq files in their version 2 (Illumina Hi-Seq) available for tissues affected by cancer or not (paired tissues), from TCGA (https://cancergenome.nih.gov/) accessed in February 2016. Version 2 gives gene expression values for 20,532 genes referred to as GeneSymbol, calculated by *RNA-seq through expectation maximization* (RSEM) (Li and Dewey, 2011) and normalized according to the upper quartile methods. The 9,190 genes for which the equivalence between GeneSymbols and UniProtKB could be obtained went through further analysis. This equivalence list is available in **Supplementary Table 1**.

The data selection followed two criteria: i) for each cancer type, a minimum of 30 patients with paired samples (control and tumor samples from the same patient) was required to satisfy statistical significance; and ii) the tumor sample had to be from a solid tumor. The data used in this work included 475 paired samples, shown in **Table 1**.

**Table 1:** Number of tumor, control, and paired samples for nine different cancer types in The Cancer Genome Atlas (TCGA) (2016).

Cancer	Tumor samples	Control samples	Paired samples
*Stomach adenocarcinoma (STAD)*	413	35	31
*Lung adenocarcinoma (LUAD)*	512	58	57
*Lung squamous cell carcinoma (LUSC)*	497	51	49
*Liver hepatocellular carcinoma (LIHC)*	738	99	48
*Kidney renal clear cell carcinoma (KIRC)*	529	72	70
*Kidney renal papillary cell carcinoma (KIRP)*	289	32	32
*Breast invasive carcinoma (BRCA)*	1,082	99	81
*Thyroid cancer (THCA)*	500	57	56
*Prostate cancer (PRAD)*	483	51	50
*Total*	5,043	554	475

The cancer molecular subtypes could be determined based on the following references: (The Cancer Genome Atlas Research Network, 2012a; The Cancer Genome Atlas Research Network, 2012b; The Cancer Genome Atlas Research Network, 2013; The Cancer Genome Atlas Research Network, 2014a; The Cancer Genome Atlas Research Network, 2014b; The Cancer Genome Atlas Research Network, 2015; Guo et al., 2016; The Cancer Genome Atlas Research Network, 2016). However, the number of paired samples in each subtype did not reach the threshold of statistical significance (n = 30), and they were therefore not considered in this paper.

### Identification of Hubs in Up-regulated Genes of Tumors

To identify genes that were significantly differentially expressed in the tumor samples of patients, we subtracted gene expression values of control samples from their respective tumor paired sample. The resulting values were called differential gene expression. Negative differential gene expression values indicated higher gene expressions in control samples, while positive differential gene expression values indicated higher gene expressions in tumor samples.

We analyzed the frequency distribution of differential gene expressions of 9,190 genes for each patient. The relative frequencies obtained, represented by *y* *_i_*, were transformed using the relationship *y’* *_i_* = log10 (*y* *_i_* + 1) and approximated to a Gaussian distribution by best fitting in GraphPad Prism software with a 95% level (Carels et al., 2015a; Carels et al., 2015b). We considered the area under the Gaussian curve to determine the one-tail threshold values that would limit *p*-values ≤ 0.05. The up-regulated genes were those with expression values above the one-tail threshold. This analysis was performed for each patient individually in R.

In a subsequent step, the PPI subnetworks were inferred for the proteins identified as products of up-regulated genes. We only considered up-regulated genes since they are those representing the tumor phenotype and the inhibition of the proteins they encode is expected to minimize potential toxic side effects to patients. The subnetworks were obtained by comparing these gene lists with the human interactome.

The human interactome was obtained from the intact-micluster.txt file (version updated December 2017) accessed on January 11, 2018, at ftp://ftp.ebi.ac.uk/pub/databases/intact/current/psimitab/intact-micluster.txt. We excluded incomplete and non-human interactions from this file, and the resulting file presented 151,631 interactions among 15,526 human proteins with UniProtKB accessions. These data can be retrieved from **Supplementary Table 2**.

We used the PPI subnetworks of up-regulated genes from each patient to identify the node degree of each protein through automated counting of their edges. These values were used to calculate the Shannon entropy of each PPI subnetwork as explained in Section “Shannon Entropy” below. In parallel, we selected the 10 proteins with the highest degree (hubs) for each patient (top-10 proteins), and we validated the five most frequent hubs among them for each tumor type regarding their biological relevance as targets through literature searches. Finally, we characterized the up-regulated genes from the MDA-MB-231 breast cancer cell line as described in Ref. (Carels et al., 2015a). Those genes were used in the PPI subnetwork construction, which was performed with the interactome and the methodology described above. The resulting subnetwork was used for Shannon entropy analysis as a reference to the extension of cell line inferences to tumor tissues as presented in this report.

### Shannon Entropy

The Shannon entropy was calculated with formula 1, where *p*(*k*) is the probability of occurrence of a vertex with a rank order *k* (*k* edges) in the subnetwork considered. The subnetworks were generated automatically from gene lists found to be up-regulated in each patient and the cell line MBA-MD-231 as described in the previous section. All operations were performed using Perl codes that can be obtained upon request.

The Shannon entropy was also used to assess the relevance of treatment directed against connection hubs by comparing the decrease in subnetwork entropy induced by hub removal with that obtained by random target selection. We randomly removed five nodes from the network and calculated the resulting entropy. This process was repeated 1,000 times for each patient to build an empirical distribution of entropies. Next, we compared the entropy found after hub removal with the distribution of entropies found after removal of nodes selected at random (see **Supplementary Table 3**).

### Overall Survival

The 5-year survival rates of the tumor tissues were inferred based on the overall survival (OS) data available from The Cancer Genome Atlas Clinical Cata Resource (TCGA-CDR) (Liu et al., 2018), which contains curated clinical and survival data from TCGA patients whose purpose was to eliminate incomplete survival (follow-up) information. **Table S1** of Liu et al. (Liu et al., 2018) has two columns, “OS” and “OS.time,” that were used in GraphPad Prism software for survival curve analysis, indicating death/event as 1 and censored data as 0. This analysis resulted in survival rates corresponding to days to “death/last follow-up” for each cancer type (**Supplementary Table 4**). The survival rate found over 5 years (in days) was used to represent each cancer type.

Finally, to determine each patient survival rate, we retrieved the number of days to “death/last follow-up” from each patient (**Supplementary Table 5**) and searched for its respective survival rate in **Supplementary Table 4**. These data were used to calculate the correlation between survival rate and entropy considering patients with the same tumor type.

### Average Target Number Per Tumor Tissue

We analyzed the subnetwork entropies of up-regulated genes associated with each cancer type and their respective 5-year survival rate. We performed a Kruskal–Wallis test and a Wilcoxon signed-rank test to determine if all samples had the same entropy average and, if not, which pairs had significantly different averages. We also analyzed the correlation coefficient between the averages of entropies per tumor type and their respective 5-year survival rate and the fitted linear regression (see **Supplementary Table 6**).

The correlation obtained between subnetwork entropies from up-regulated genes and survival rates allowed the inference of the approximate number of targets to be inactivated for each patient. The 20 proteins from the up-regulated subnetwork with the largest connection counts were called top-20 targets. In order to mimic the effect of inhibiting top-1 to top-20 targets, we excluded each target from the patient subnetwork of up-regulated genes. The Shannon entropy was calculated for the resulting subnetworks, and the suitable number of hubs for inactivation was found when the entropy of top-n subnetworks was equal to or less than the entropy that would correspond to the 100% survival rate (see **Supplementary Table 7**).

In addition, we performed the same experiments considering the suitable number of hubs for inactivation and the entropy found for patients in a given tumor type. In this case, we selected tumor types with at least three patients with “death/last follow-up” in each survival rate interval (100%–81%, 80%–61%, 60%–41%, 40%–21%) to calculate the entropy and survival rate averages. Only LUAD and LUSC satisfied these requirements (**Supplementary Table 5**).

## Results

### Identification of Up-regulated Hubs in Patients

We identified 273 proteins among all hub combinations of top-10 proteins for each patient. From those proteins, 112 (41.0%) were patient specific, 143 (52.4%) were specific for one cancer type, and only 16 (5.9%) were found in combinations over every cancer type. Furthermore, only four patients shared the same top-10 combination, two from BRCA and two from PRAD. This means that 99% of patients had a unique combination of top-10 hubs, even if some hubs could be found conserved across a significant part of the patient population. This property can be related to the variation in the number of connections for each hub according to the patients’ subnetwork of up-regulated genes and to the variation of hubs that are up-regulated from one tumor to the other. The hub combination for each patient and their respective connection number in each subnetwork are given in **Supplementary Table 8**.

The five most frequent hubs among patients from each cancer type are listed in **Table 2** in association with their cancer hallmarks and the respective literature reference.

**Table 2:** List of the five most frequent hubs, their incidence in patients for each cancer type, and their respective cancer hallmark.

Cancer type	Hub	Number of patients	Percentage of patients	Cancer hallmark	Reference
STAD	HSP90AB1	29	90.6%	Genomic instability	(Haase and Fitze, 2016)
	MYH9	27	84.3%	Cell adhesion, invasion and migration	(Li and Yang, 2016)
	YWHAZ	22	68.7%	Cell proliferation, invasion and migration	(Nishimura et al., 2013; Deng et al., 2019; Hong et al., 2018)
	FN1	18	56.2%	Cell adhesion, invasion and migration, cell growth, cell death escape	(Soikkeli et al., 2010; Wang et al., 2017)
	HSPA5	14	43.7%	Genomic instability	(Haase and Fitze, 2016)
LUAD	YWHAZ	48	84.2%	Cell proliferation, invasion and migration	(Nishimura et al., 2013; Deng et al., 2019; Hong et al., 2018)
	HSP90AB1	46	80.7%	Genomic instability	(Haase and Fitze, 2016)
	HSPA5	38	66.6%	Genomic instability	(Haase and Fitze, 2016)
	FN1	30	52.6%	Cell adhesion, invasion and migration, cell growth, cell death escape	(Wang et al., 2017; Soikkeli et al., 2010)
	ACTB	27	47.4%	Invasion and migration	(Guo et al., 2013)
LUSC	YWHAZ	48	96.0%	Cell proliferation, invasion and migration	(Nishimura et al., 2013; Deng et al., 2019; Hong et al., 2018)
	HSP90AB1	39	78.0%	Genomic instability	(Haase and Fitze, 2016)
	HSPA5	28	56.0%	Genomic instability	(Haase and Fitze, 2016)
	TP63	25	56.0%	Cell growth	(Jiang et al., 2018)
	NPM1	21	44.0%	Invasion and migration, inflammation, genomic instability	(Loubeau et al., 2014; Box et al., 2016; Lin et al., 2016)
LIHC	HSP90AB1	41	83.6%	Genomic instability	(Haase and Fitze, 2016)
	HSPB1	28	57.1%	Genomic instability, cell death escape	(Deng et al., 2019; Hong et al., 2018)
	MYH9	27	55.1%	Cell adhesion, invasion and migration	(Li and Yang, 2016)
	ACTB	24	48.9%	Invasion and migration	(Guo et al., 2013)
	YWHAZ	21	42.9%	Cell proliferation, invasion and migration	(Nishimura et al., 2013; Deng et al., 2019; Hong et al., 2018)
KIRC	FN1	61	87.1%	Cell adhesion, invasion and migration, cell growth, cell death escape	(Wang et al., 2017; Soikkeli et al., 2010)
	RPL10	56	80.0%	Invasion and migration, cell death escape	(Goudarzi and Lindström, 2016; Shi et al., 2018)
	VCAM1	55	78.6%	Inflammatory response, cell adhesion, cell growth	(Huang et al., 2013; Kong et al., 2018)
	NPM1	47	67.1%	Invasion and migration, inflammation, genomic instability	(Loubeau et al., 2014; Box et al., 2016; Lin et al., 2016)
	ACTB	43	61.4%	Invasion and migration	(Guo et al., 2013)
KIRP	ACTB	25	78.1%	Invasion and migration	(Guo et al., 2013)
	LRRK2	20	62.5%	Cell proliferation, cell death escape	(Wallings et al., 2015)
	VCAM1	18	56.3%	Inflammatory response, cell adhesion, cell growth	(Huang et al., 2013; Kong et al., 2018)
	FN1	17	53.1%	Cell adhesion, invasion and migration, cell growth, cell death escape	(Soikkeli et al., 2010; Wang et al., 2017)
	HSPB1	16	50.0%	Genomic instability, cell death escape	(Deng et al., 2019; Hong et al., 2018)
BRCA	FN1	80	95.2%	Cell adhesion, invasion and migration, cell growth, cell death escape	(Wang et al., 2017; Soikkeli et al., 2010)
	ACTB	58	69.0%	Invasion and migration	(Guo et al., 2013)
	YWHAZ	55	65.5%	Cell proliferation, invasion and migration	(Nishimura et al., 2013; Deng et al., 2019; Hong et al., 2018)
	HSP90AB1	49	58.3%	Genomic instability	(Haase and Fitze, 2016)
	ESR1	44	52.3%	Cell proliferation, invasion and migration, escape immune response	(Mostafa et al., 2014; Ahmed and Aboelnaga, 2015; Jeselsohn et al., 2015)
THCA	FN1	47	83.9%	Cell adhesion, invasion and migration, cell growth, cell death escape	(Wang et al., 2017; Soikkeli et al., 2010)
	LRRK2	41	73.2%	Cell proliferation, cell death escape	(Wallings et al., 2015)
	FLNA	39	69.6%	Cell adhesion, invasion and migration, growth, cell death escape	(Shao et al., 2016)
	RPL10	27	48.2%	Invasion and migration, cell death escape	(Shi et al., 2018; Goudarzi and Lindström, 2016)
	MYH9	25	44.6%	Cell adhesion, invasion and migration	(Li and Yang, 2016)
PRAD	HSPA5	31	62.0%	Genomic instability	(Haase and Fitze, 2016)
	HSP90AB1	30	60.0%	Genomic instability	(Haase and Fitze, 2016)
	RPL10	30	60.0%	Invasion and migration, cell death escape	(Goudarzi and Lindström, 2016; Shi et al., 2018)
	NPM1	29	58.0%	Invasion and migration, inflammation, genomic instability	(Loubeau et al., 2014; Box et al., 2016; Lin et al., 2016)
	NDRG1	28	56.0%	Invasion and migration, growth	(Menezes et al., 2017)

The heat shock protein AB1 (HSP90AB1) was identified in 65% of all patients and is the most common hub identified among all 475 patients. High expression of HSP90AB1 has been associated with aggressive phenotypes in HER2-negative breast cancer (Cheng et al., 2012), and it is also identified as a hub target for MDA-MB-231, a triple-negative breast cell line. Its inhibition in combination with four other hubs decreased cell growth, proliferation, migration, and invasion *in vitro* (Tilli et al., 2016). **Table 2** also shows two other heat shock proteins: HSPA5 and HSPB1. Since heat shock proteins function as chaperones, they are essential for cell maintenance and survival. Their relationship with tumor development is associated with their ability to stabilize mutant proteins, resulting from increased genomic instability, which would be degraded without the chaperones’ assistance (Haase and Fitze, 2016). Specifically, HSPA5 has been identified in 38.7% of patients belonging to all nine cancer types, while HSPB1 has been identified in 27.2% of patients distributed among eight cancer types. The latter protein has been described as responsible for cancer escaping cell death and has been proposed as a specific biomarker for monitoring ovarian cancer patients’ response to chemotherapy (Sun et al., 2015; Stope et al., 2016).

Fibronectin 1 (FN1) is found in 64% of patient combinations belonging to all nine cancer types analyzed. FN1 has been widely described in tumor progression and is a member of multiple hallmarks, such as cell adhesion, invasion, migration, growth, and cell death escape (Soikkeli et al., 2010; Wang et al., 2017). Its high expression level has been associated with increased aggressiveness in thyroid cancer (Sponziello et al., 2016), and its expression in renal clear carcinoma is associated with a higher disease-related mortality rate (Steffens et al., 2012). Furthermore, its inactivation through microRNA has inhibited papillary thyroid carcinoma progression (Ye et al., 2017).

The tyrosine 3-monooxygenase/tryptophan 5-monooxygenase activation protein zeta (YWHAZ) was identified in 55.9% of all patients’ combinations from all nine cancer types. This protein is present in high levels in different cancer cells and is associated with tumor cell proliferation, cell invasion and migration, and drug resistance (Nishimura et al., 2013; Hong et al., 2018; Deng et al., 2019). This protein target has prognostic potential, and its overexpression is associated with short OS time in non-squamous-cell lung carcinoma (Deng et al., 2019); its knockdown has suppressed tumorigenesis in ovarian cancer cells (Hong et al., 2018).

**Figure 1:**
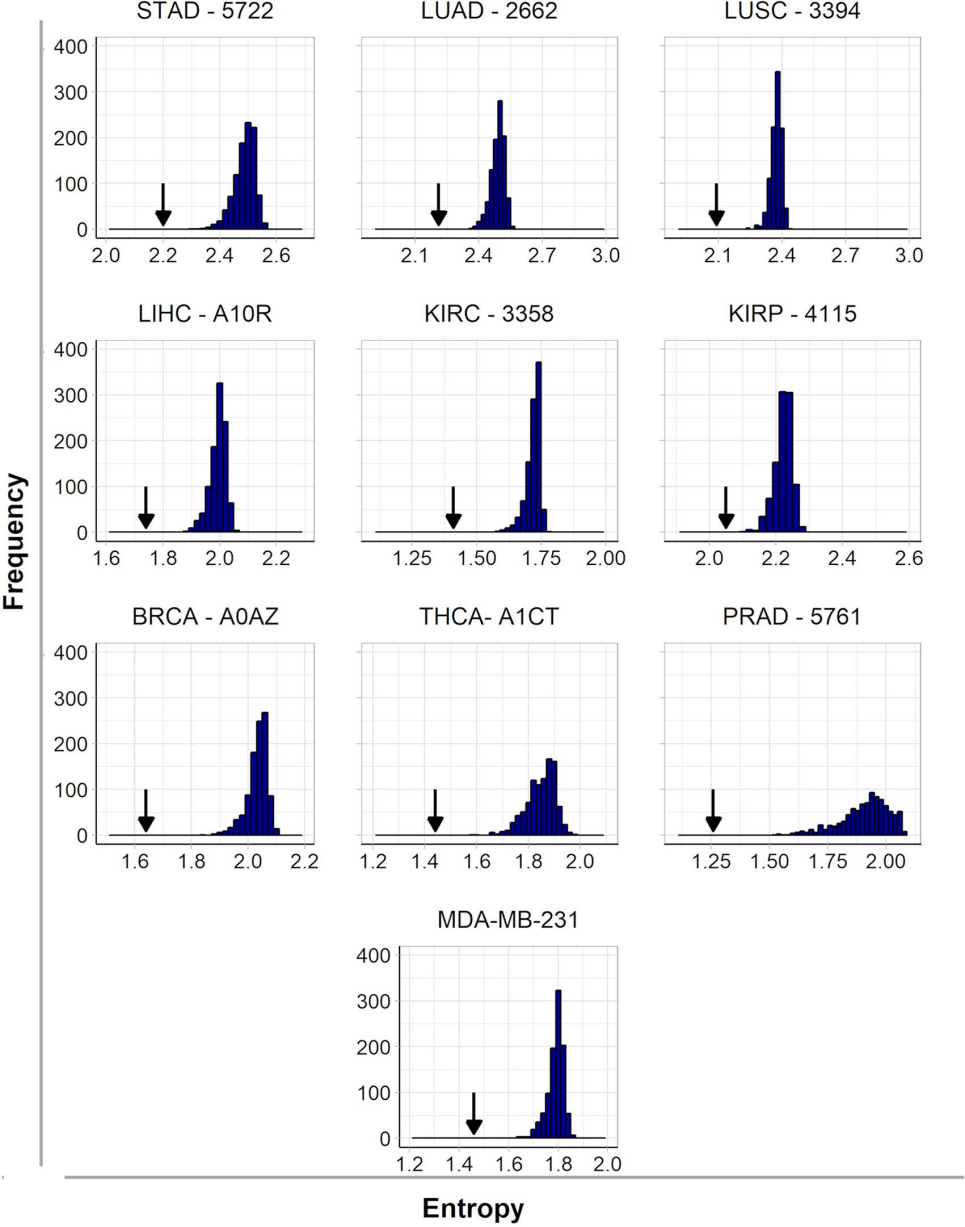
Histograms of frequency and entropy distribution after inactivation of targets selected at random from subnetworks of up-regulated genes from nine patients (one from each cancer type) and the cell line MDA-MB-231. The arrows indicate the entropy found after hubs’ inactivation from the same subnetwork.

It is interesting to note here that we found some common targets between tumors and cell lines, which is expected since the origin of malignant cell lines is a tumor sample. However, the treatment of tumors is more complex because it is heterogeneous and formed by several cell lines. For this reason, the protein targets identified for each patient are encoded by up-regulated genes identified by comparison to their paired control since the RNA-seq of a tumor cannot differentiate the various cell lines that compose this tumor. In order to assess the effect of inhibiting selected hubs on tumor subnetwork entropy, we compared it to the effect that would be obtained inhibiting randomly selected targets in these subnetworks. Indeed, the inactivation of five hubs had a significantly higher effect in decreasing entropy than the inactivation of five targets selected at random. One example for each cancer type is given in **Figure 1**, and all results are given in more detail in **Supplementary Table 3**. These results indicate that the entropy increase in cancer is driven mainly by hubs (Teschendorff et al., 2015), which has the corollary of an increase in the number of alternative pathways in more aggressive tumors.

The effect of disrupting tumor subnetworks by hub inactivation is similar to the one obtained with the subnetwork of MDA-MB-231 (**Figure 1**). Indeed, as discussed in the introduction, the simultaneous inactivation of the top-5 hubs identified for this cell line resulted in significant reduction of tumor activity without any side effects to a non-tumoral cell line used as a control (Tilli et al., 2016). This result suggests that our strategy should be as successful in heterogeneous tumor samples as it was in cell lines. In this sense, this report is a generalization to tumors of our entropy-based strategy formally established with cell lines. Since such a strategy is not obvious a priori, it may be considered as a significant progress for translational medicine because it enables to us infer rational strategies for cancer therapies by personalized approaches.

### Tumor Entropy and Its Correlation With Overall Patient Survival Rate

We considered the subnetworks of up-regulated genes to calculate the Shannon entropy relative to the tumor sample of each patient and used the averages to represent each cancer type. The number of genes up-regulated in tumors varied from patient to patient, but the average number for each cancer type was between 250 and 450. A supplementary table shows the number of up-regulated genes and the entropy calculated for each patient (see **Supplementary Table 6**).

The entropy found for the subnetworks of up-regulated genes was used to analyze tumor complexity and its relationship with OS rate. The OS data available in ref. (Liu et al., 2018), which contains curated clinical and survival data from TCGA patients, were used to infer the 5-year survival rate for each cancer type (for more details, see “*Materials and Methods*”).

**Figure 2:**
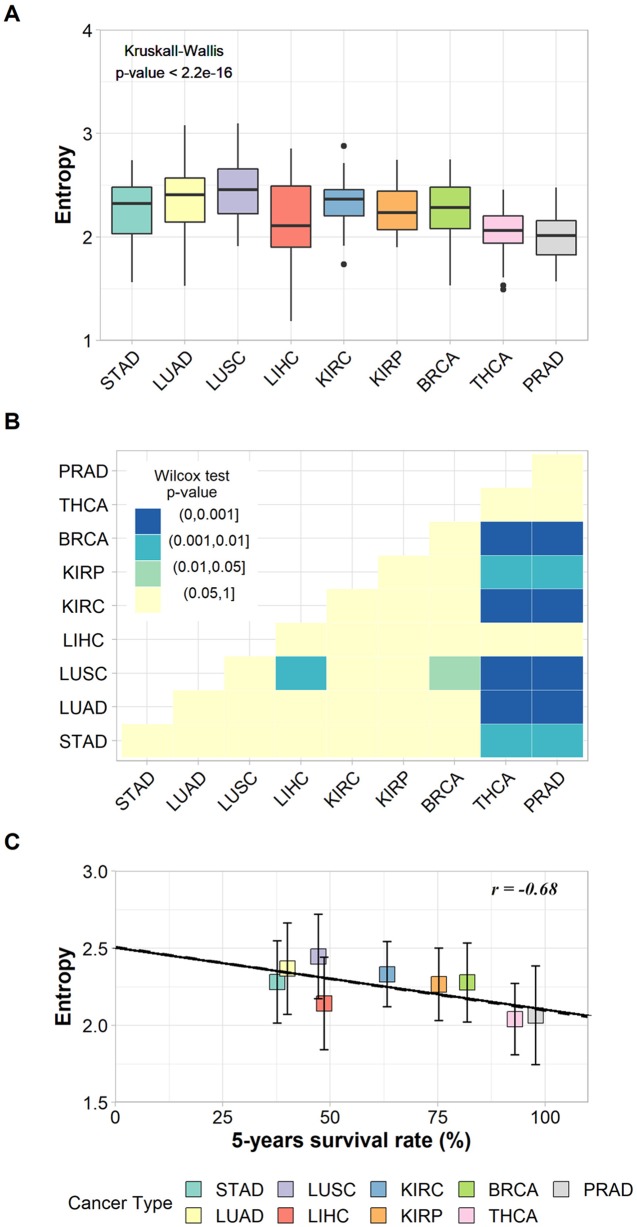
**(A)** Box plot of the entropies found for the subnetworks of up-regulated genes in each cancer type and the result of Kruskal–Wallis test. **(B)** Heat map of *p*-values from Wilcoxon pairwise comparison. Light-yellow squares represent non-significant *p*-values; other colors represent significant but different *p*-values according to legend. **(C)** Correlation between entropies of up-regulated genes’ subnetworks and their respective 5-year survival rates. The vertical bars indicate standard deviations.

The non-parametric Kruskal–Wallis test was performed to assess whether the entropy averages were the same for all cancer types. We found a chi-squared value of 94.9, degrees of freedom = 8, and a *p*-value < 2.2e−16, which refutes the null hypothesis of average entropy equality and indicates that at least one cancer type has an average significantly different from another one (**Figure 2A**). The Wilcoxon pairwise comparison test confirmed that cancer types with large differences in survival rates have significant average entropy differences (**Figure 2B**). For instance, **Figure 2B** shows that thyroid cancer (THCA) and prostate cancer (PRAD), the cancer types with the highest survival rates, have significantly different entropy averages in comparison to almost all other cancer types, with the exception of LIHC. In addition, LUSC also has significantly different entropy averages when compared to BRCA and LIHC (**Figure 2B**). The *p*-values found for each pairwise analysis can be found in **Supplementary Table 9**.

The relationship between tumor entropy and 5-year survival rates was characterized by a negative correlation (*r* = −0.68, *p*-value = 0.043), and the fitted regression line (Y = −0.004X + 2.507) had a slope within a 95% confidence interval from −0.00800 to −0.00017 (**Figure 2C**). These results indicate that the regression line was significantly different from the horizontal (slope = 0.00000).

These results were based on patient data and are in agreement with those proposed elsewhere (Teschendorff and Severini, 2010; van Wieringen and van der Vaart, 2011; Breitkreutz et al., 2012; West et al., 2012; Banerji et al., 2015), indicating that the subnetwork entropy of tumor tissues increases together with the tumor aggressiveness.

The linear regression indicates a common trend between malignancy and entropy for each tumor. However, the standard deviations associated with each tissue’s averages indicate a variation in entropy between patients with the same cancer type, which means that despite sharing some common molecular features, each patient has his/her own tumor complexity and aggressiveness.

The relationship between tumor entropy and survival rates also holds when considering patients from the same tumor type. For LUAD, we found a significant negative correlation of −0.98 between entropy average and survival rate, with a significant *p*-value = 0.02 and a slope in the 95% confidence interval from −0.005 to −0.001. For LUSC, we found a negative correlation of −0.70, with a non-significant *p*-value = 0.29 and a slope in the 95% confidence interval from −0.015 to 0.007. The lack of statistical significance is most likely due to the small sample size (n = 4). All these data can be seen in more detail in **Supplementary Tables 5** and **6**.

The limitation in the tissue diversity of our report is justified by statistical constraints. However, this tissue diversity covers the whole range of tumor aggressiveness. The small slopes found in the regression analyses (found across patients with tumors in multiple tissues and within the set of patients with tumors in the same tissue, such as LUAD and LUSC) can be explained by the use of Shannon entropy as a complexity measure, whose values vary in the first decimal between 2.0 and 2.5 for patients between 35% and 100% survival, on the average. This approach requires a significant difference of survival rates between patients with different tumor types or patients within one tumor type to reveal a significant difference in their respective entropies. For this reason, it was not possible to analyze patients among PRAD and THCA. Both included patients with high survival rates, between 100.0% and 93.0% for PRAD, and from 99.7% to 89.6% for THCA. Furthermore, the other tissues (kidney renal clear cell carcinoma -KIRC, kidney renal papillary cell carcinoma - KIRP and stomach adenocarcinoma – STAD) had only three survival rate intervals available, which is not enough to generate a statistically significant regression line.

Moreover, correlations between subnetwork size and entropy (*r = *−0.12, *p*-value = 0.74) or between subnetwork size and survival rates (*r = *0.48, *p*-value = 0.18) were not large enough and not even statistically significant to suggest any linear relationship.

Many efforts were made for stratifying patients based on their molecular subtypes that would suggest better patient treatment (The Cancer Genome Atlas Research Network, 2012a; The Cancer Genome Atlas Research Network, 2012b; The Cancer Genome Atlas Research Network, 2013; The Cancer Genome Atlas Research Network, 2014a; The Cancer Genome Atlas Research Network, 2014b; The Cancer Genome Atlas Research Network, 2015; Guo et al., 2016; The Cancer Genome Atlas Research Network, 2016). Unfortunately, due to the small number of patients (n∼3) with paired samples characterized in each subtype and the few subtypes described in each tumor type (∼2), we could not explore the potential differences in entropy and survival rates at the subtype level.

### Identification of Hubs Whose Inhibition Could Benefit Patients

Our results suggest a prognostic potential of the entropy measure: the higher the entropy, the worse the prognosis. This relationship was previously described for the patient outcome according to breast cancer major subtypes and lung cancer (Banerji et al., 2015). Therefore, reducing the subnetwork entropy by inhibiting the most connected hubs encoded by up-regulated genes should improve the patient’s prognosis.

As indicated above, each tumor has a specific entropy value around the mean of its corresponding tissue. For this reason, the question is how much one should decrease the entropy value on a case-to-case basis in order to improve the patient’s prognosis and treatment benefit. In this context, we hypothesized that decreasing the entropy value to the one corresponding to 100% survival rate, we would increase the patient’s prognosis by a signaling network restructuring specific to tumor tissues, which is in line with the *in vitro* validation performed earlier (Tilli et al., 2016).

When we compared the tumor entropies found for each patient to the entropy corresponding to 100% survival, we found out that 318 of them would need at least one hub inhibition. The remaining patients showed tumor entropies lower than the one expected for 100% survival. This unexpected behavior could be explained by two biological facts: signaling heterogeneity, which may occur in early tumor stages when the difference in cell differentiation between normal and tumor tissues is still very small, and sampling heterogeneity, when surrounding tumor tissues, considered as control samples in this work, are invaded by cancer stem cells (Lau et al., 2017). The entropy of cancer stem cells has been previously described as higher than that of differentiated normal or tumor cells due to their larger signaling pathway heterogeneity (Banerji et al., 2013), which may explain why the resulting entropy for these patients is lower than the one expected for 100% survival rate.

**Figure 3:**
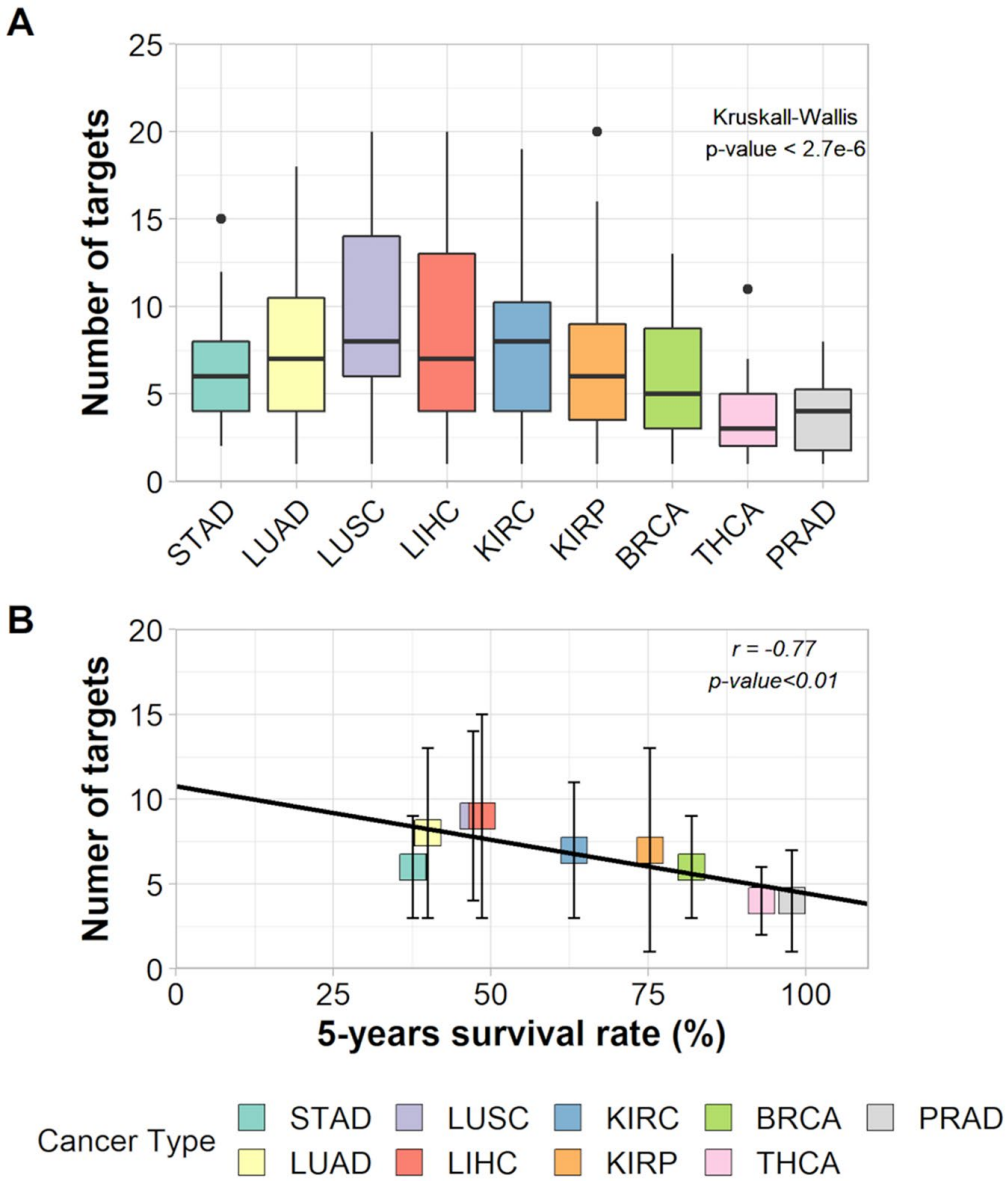
**(A)** Box plot of the number of targets in each cancer type and the result of Kruskall–Wallis test. **(B)** Correlation between number of targets and 5-years survival rates of each cancer type.

The Kruskal–Wallis test and a Wilcoxon pairwise comparison considering the number of targets indicated for inhibition show a significant difference between tissues averages (**Figure 3A** and **Supplementary Table 10**). The relationship between the number of targets and 5-year survival rates was characterized by a negative correlation (*r* = −0.77, *p*-value < 0.011) and had a slope = −0.067 within a 95% confidence interval from −0.114 to −0.020 (**Figure 3B**). This result suggests that, on average, cancer types with lower survival rates need a higher number of hubs for inhibition (around eight) than cancer types with higher survival rates (around four hubs).

Yet, the selection of as many targets as necessary to decrease the tumor entropy level to that of 100% patient survival showed that patients from all nine cancer types needed the inhibition of target combinations varying from 1 to 20 hubs. The effect on entropy and the number of targets to be inhibited for each patient is given in **Supplementary Table 7**.

## Discussion

Many efforts were made to better understand cancer through Shannon entropy approaches. We confirmed here the existence of a significant negative correlation between the entropy of up-regulated gene subnetworks and patients’ survival rate in real cases, which suggests a larger ability of aggressive tumors to switch among alternative pathways and to overcome environmental challenges such as drug treatments.

### The Relevance of Targeting Connection Hubs

The decrease of entropy values after hub inactivation was significantly higher than the decrease of entropy after inactivation of targets selected at random, which confirms the benefit of a targeted attack on scale-free systems as shown by Albert et al. (Albert et al., 2000). The success of this approach has been validated in cell lines *in vitro *(Tilli et al., 2016). The relevance of targeting hubs allows us to investigate the quality (nature) and the quantity (number) of hubs that should be taken into consideration to rationally design a drug cocktail for a patient with a given gene expression profile. For this purpose, we investigated the number of connectivity hubs indicated for inhibition according to tumor aggressiveness using Shannon entropy. The benefit of characterizing a topology using entropy is based on the fact that this measure is invariant with respect to network size since the entropy calculation involves probabilities rather than absolute values. This concept is important in the context of this study because the subnetworks investigated were varying in size even if the criteria for their construction were kept identical from one patient to another.

### Shannon Entropy and Tumor Aggressiveness

Our results are in accordance with previously published works that also describe a negative correlation between tumor entropy and aggressiveness. For instance, Breitkreutz et al. considered entropy as a tumor complexity measure and found an *r* ^2^ = 0.7 between the Shannon entropy of tumors and the 5-year survival rate after treatment, which means that 70% of the variance among the 14 samples considered was well represented by linear regression (Breitkreutz et al., 2012). Despite these outstanding results, the reference to KEGG regulatory pathways (http://www.genome.jp/kegg/) can be argued as non-representative of the real cases since these pathways include only a few vertices, ranging from 25 to 50, and are constructed as a consensus based on the data from many patients. Obviously, tumors are more complex and may present variations of the KEGG patterns of regulatory pathway dysregulation between patients with the same cancer type.

In addition, entropy was used as a measure of system randomness (Teschendorff and Severini, 2010), system disorder (West et al., 2012), and heterogeneity in order to quantify the network signaling rather than network topology (Banerji et al., 2015). All these works found higher entropies for cancer samples than control samples. Larger entropies were also associated with metastatic compared to non-metastatic tumors and also with advanced stages compared to early tumor stages (van Wieringen and van der Vaart, 2011).

All these methodologies considered a unique signaling network with different interaction weights assigned according to the phenotypic expression data. In a similar way, we took expression data into consideration in our analysis but through the determination of expression thresholds above which genes would have to be considered as up-regulated. This *binary* approach of incorporating expression data into PPI directly affects the features of subnetworks regarding their topology.

### Identification of Molecular Targets

We found that despite using the same protocol to select up-regulated genes and the same interactome, all subnetworks had different sizes and hub profiling. The variation of Shannon entropy among patients of the same tumor type indicates how much the individual profiling of molecular targets is recommended for rational therapeutic design.

As shown here, the transcriptome and PPI data currently available allow the development of personalized medicine, which offers the possibility of a rational therapeutic approach based on individual molecular data, i.e., maximizing the patient benefit of therapy by designing the best combination of targets to be inhibited through personalized cocktails of drugs and/or biopharmaceuticals. Furthermore, the inhibition of hubs preferentially expressed in tumor samples would minimize overlapping toxicity effects.

The reference to the entropy corresponding to 100% patient survival enabled us to explore putative individual therapeutics as well. Despite the general trend of higher entropy associated with aggressive tumors, each patient would need the inhibition of a different hub combination to reach the entropy level corresponding to 100% patient survival.

These ideas were first proposed through bioinformatic inference with cell line expression data (Carels et al., 2015a) and then validated *in vitro* (Tilli et al., 2016). Here, we extended these concepts to their application to patient data as an initial step toward translational medicine. Our approach was shown to be robust once the hubs identified were validated in the literature as key players in the processes known to be key for tumor development.

### Future Direction

The large target number between 10 and 20 necessary in some patients to reduce the entropy of the tumor tissue to the level corresponding to the 100% patient survival probability may be a consequence of our approach. The effects of hub inactivation were analyzed considering a static network, in which we had to implement all interventions in order to reach the desired state. However, the signaling network is dynamic due to regulatory interactions between proteins and genes, and it is possible that the inhibition of a smaller target set may trigger a cascade effect resulting in irreversible tumor cell death as suggested by Tilli et al. (Tilli et al., 2016).

In this context, each gene expression profile of the signaling network may be interpreted as a state. The set of all states represents a continuous and multidimensional state space defined by the number of genes analyzed and their expression. Some states may define a cell phenotype or correspond to cell differentiation. Those states can be called attractors and are characterized as an equilibrium point in phase space. However, due to the stochastic nature of gene regulation, other states might result in the same phenotype as the attractor, and for this reason, they form the basin of attraction (Huang et al., 2009).

Once we consider cancer disease as resulting from attractors in the phase space of cellular dynamics, therapy should lead the signaling network toward a new basin of attraction of active cell death (Cornelius et al., 2013; Huang et al., 2009). The analysis of the signaling network state space should allow the identification of the basin related to the desired attractor, optimizing the number of targets indicated for treatment. Moreover, this strategy would also allow the identification of an order of priority for therapeutic interventions required to reach the basin of attraction related to the desired state (Cornelius et al., 2013).

Also, we assumed here that any target can be inhibited in different ways, using drugs, aptamers, interference RNA, or other methodologies that will have different consequences for patients depending on their off-target activity. This exercise also assumes that the inhibition of any target combination is possible, without considering interactions between drugs or any other side effect of a pharmacokinetic nature (differences in drug metabolism among human haplotypes).

Therefore, our approach still requires a case-by-case examination according to additional layers of complexity associated with the dynamics of signaling networks and methods for target inactivation.

## Conclusion

Topological measures of PPI networks bring useful information for personalized treatment of cancer. Among the measures of node and path metrics, we focused this study on the application of Shannon entropy to subnetworks of tumors’ up-regulated genes. The results of our analysis in this paper show the following: (i) As proposed by Albert et al. (Albert et al., 2000), our experiment showed that removing the most connected targets is more effective than removing targets at random, whatever their connectivity degree. (ii) The gross approximation by Breitkreutz et al. (Azevedo and Moreira-Filho, 2015) is confirmed using interactome and RNA-seq data of real tumors, but the slope of the regression line obtained is lower than that published by these authors. This shows the need for large changes in network complexity to observe a difference in survival rates. (iii) As expected from the intra-tumor heterogeneity in cell line composition, we found a large standard deviation of entropy by tissue. The highly personalized molecular profile of tumors justifies an individual diagnostic and therapeutic (theranostics) approach in order to reduce toxic side effects of treatment to patients. (iv) When considering 100% survival as the goal of the treatment, the negative correlation indicates that aggressive tumors will need a larger set of therapeutic agents (drugs and/or biopharmaceuticals) than benign ones, on average, to reduce the entropy of the subnetwork of up-regulated genes to achieve a higher life expectancy.

## Data Availability Statement

All data sets generated and analyzed for this study are included in the manuscript/**Supplementary Files**.

## Author Contributions

AC participated in the conception, design, analysis, and interpretation of the work. JT participated in the conception, design, and drafting of the work. FS participated in the design of the work and substantially revised it. NC participated in the conception, design, analysis, and interpretation of the work and substantially revised it. All authors participated in the report writing and approved the final version.

## Funding

This study was supported by a fellowship from Fundação de Amparo à Pesquisa do Estado do Rio de Janeiro (FAPERJ) to AC. JT acknowledges research support from Natural Sciences and Engineering Research Council (NSERC) (Canada), the Alberta Cancer Foundation, and the Allard Foundation.

## Conflict of Interest

The authors declare that the research was conducted in the absence of any commercial or financial relationships that could be construed as a potential conflict of interest.
